# The genome sequence of the lesser sand-eel, *Ammodytes marinus *Raitt, 1934

**DOI:** 10.12688/wellcomeopenres.23679.2

**Published:** 2025-10-06

**Authors:** Helga Bára Mohr Vang, Ian Salter, Svein-Ole Mikalsen, Sunnvør í Kongsstovu, Wellcome Sanger Institute Tree of Life Management, Samples and Laboratory team, Wellcome Sanger Institute Scientific Operations: Sequencing Operations, Wellcome Sanger Institute Tree of Life Core Informatics team, Tree of Life Core Informatics collective

**Affiliations:** 1 Faroe Marine Research Institute, Tórshavn, Streymoy, Faroe Islands; 2 Faculty of Science and Technology, University of the Faroe Islands, Tórshavn, Faroe Islands

**Keywords:** Ammodytes marinus, lesser sand-eel, genome sequence, chromosomal, Uranoscopiformes

## Abstract

We present a genome assembly from a specimen of *Ammodytes marinus* (the lesser sandeel; Chordata; Actinopteri; Uranoscopiformes; Ammodytidae). The genome sequence has a total length of 777.80 megabases. Most of the primary assembly (95.51%) is scaffolded into 25 chromosomal pseudomolecules. The mitochondrial genome has also been assembled and is 16.53 kilobases in length. Gene annotation of this assembly on Ensembl identified 22,410 protein-coding genes.

## Species taxonomy

Eukaryota; Opisthokonta; Metazoa; Eumetazoa; Bilateria; Deuterostomia; Chordata; Craniata; Vertebrata; Gnathostomata; Teleostomi; Euteleostomi; Actinopterygii; Actinopteri; Neopterygii; Teleostei; Osteoglossocephalai; Clupeocephala; Euteleosteomorpha; Neoteleostei; Eurypterygia; Ctenosquamata; Acanthomorphata; Euacanthomorphacea; Percomorphaceae; Eupercaria; Uranoscopiformes; Ammodytidae; *Ammodytes*; *Ammodytes marinus* Raitt, 1934 (NCBI:txid146480).

## Background

**Figure 1.**
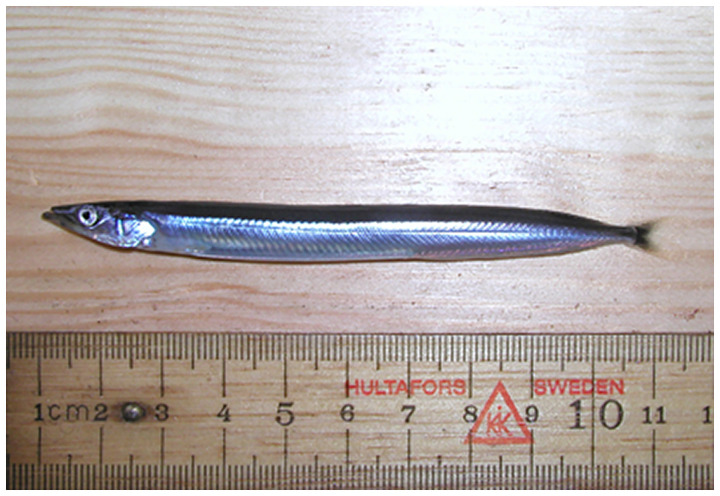
*Ammodytes marinus* individual sampled by the Faroe Marine Research Institute, with a scale in inches. (not the specimen used for genome sequencing).

The lesser sandeel ( *Ammodytes marinus*) is a small fish characterised by its elongated body, pointed head, and silvery colouration ( Figure 1). It typically grows to a length of about 25 cm and has a lifespan of 5 to 10 years ( Mouritsen, 2007; Muus & Nielsen, 1999). This species belongs to a group of closely related fishes collectively referred to as sandeels ( *Ammodytes* spp. and *Hyperoplus* spp.). Sandeels are known to burrow into sandy seabeds for protection at night and during the winter months. During the day, they are benthopelagic and feed primarily on plankton ( Winslade, 1974). The various sandeel species have overlapping geographical distributions, with the lesser sandeel spanning much of the Northeast Atlantic. Its range extends from 49°N to 74°N, covering areas from eastern Greenland to the Baltic Sea ( Mouritsen, 2007).

Sandeels are cornerstone species in costal and shallow marine ecosystems, playing a vital role as prey for seabirds, larger fish, and marine mammals. As such they serve as a critical link between primary production and higher trophic levels ( Eliasen *et al.*, 2011). Humans also exploit sandeel species, primarily for producing fishmeal and fish oil, with annual catches mounting up to 1 million tonnes ( ICES, 2017). However, fisheries activities and climate change pose significant threats to sandeel populations, emphasising the urgency of conservation measures ( MacDonald *et al.*, 2019). Reflecting these ecological concerns, the UK government implemented a prohibition on sandeel fishing in English and Scottish waters starting in March 2024 ( Government Digital Service, 2024; Scottish Government, 2024).

A chromosomal-level genome assembly of the lesser sandeel genome provides a crucial resource for conservation and research. This genetic tool represents a significant advancement in understanding the genetic health and population structure of the lesser sandeel, both of which are essential for informed conservation strategies and the sustainable management of fisheries. Moreover, genome assemblies from the lesser sandeel and closely related species will enable the precise differentiation of species providing deeper insights into their ecological roles and interactions within marine ecosystems. Such knowledge is essential for ensuring the health and resilience of marine ecosystems.

## Genome sequence report

The genome of a specimen of *Ammodytes marinus* was sequenced using Pacific Biosciences single-molecule HiFi long reads, generating a total of 42.43 Gb (gigabases) from 4.19 million reads, providing an estimated 58-fold coverage. Chromosome conformation Hi-C sequencing produced 116.36 Gb from 770.57 million reads. Specimen and sequencing details are summarised in Table 1.

**Table 1.** Specimen and sequencing data for *Ammodytes marinus*.

Project information			
**Study title**	Ammodytes marinus (lesser sand-eel)		
**Umbrella BioProject**	PRJEB60704		
**Species**	*Ammodytes marinus*		
**BioSample**	SAMEA110137622		
**NCBI taxonomy ID**	146480		
Specimen information			
**Technology**	**ToLID**	**BioSample accession**	**Organism part**
**PacBio long read sequencing**	fAmmMar1	SAMEA110137625	Gill
**Hi-C sequencing**	fAmmMar1	SAMEA110137631	Fin
**RNA sequencing**	fAmmMar1	SAMEA110137632	Fin
Sequencing information			
**Platform**	**Run accession**	**Read count**	**Base count (Gb)**
**Hi-C Illumina NovaSeq 6000**	ERR11040187	7.71e+08	116.36
**PacBio Sequel IIe**	ERR11029695	1.87e+06	18.8
**PacBio Sequel IIe**	ERR11029696	2.32e+06	23.63
**RNA Illumina NovaSeq 6000**	ERR11042966	6.47e+07	9.77

Assembly errors were corrected by manual curation, including 68 missing joins or mis-joins and eight haplotypic duplications. This reduced the scaffold number by 3.89%. The final assembly has a total length of 777.80 Mb in 616 sequence scaffolds, with 1,017 gaps and a scaffold N50 of 31.8 Mb ( Table 2).

**Table 2.** Genome assembly data for *Ammodytes marinus*, fAmmMar1.1.

Genome assembly		
Assembly name	fAmmMar1.1	
Assembly accession	GCA_949987685.1	
*Accession of alternate haplotype*	*GCA_950004245.1*	
Span (Mb)	777.80	
Number of contigs	1,634	
Number of scaffolds	616	
Longest scaffold (Mb)	39.74	
Assembly metrics *		*Benchmark*
Contig N50 length (Mb)	2.0	*≥ 1 Mb*
Scaffold N50 length (Mb)	31.8	*= chromosome N50*
Consensus quality (QV)	53.7	*≥ 40*
*k*-mer completeness	Primary: 81.08%; alternate: 79.48%; combined: 98.50%	*≥ 95%*
BUSCO **	C:98.0%[S:97.1%,D:0.9%], F:0.3%,M:1.7%,n:3,640	*S > 90%, D < 5%*
Percentage of assembly mapped to chromosomes	95.51%	*≥ 90%*
Sex chromosomes	Not identified	*localised homologous pairs*
Organelles	Mitochondrial genome: 16.53 kb	*complete single alleles*
Genome annotation of assembly GCA_949987685.1 at Ensembl		
Number of protein-coding genes	22,410	
Number of non-coding genes	2,954	
Number of gene transcripts	47,704	

* Assembly metric benchmarks are adapted from Rhie *et al.* (2021) and the Earth BioGenome Project Report on Assembly Standards September 2024. ** BUSCO scores based on the actinopterygii_odb10 BUSCO set using version 5.3.2. C = complete [S = single copy, D = duplicated], F = fragmented, M = missing, n = number of orthologues in comparison. A full set of BUSCO scores is available at https://blobtoolkit.genomehubs.org/view/fAmmMar1_1/dataset/fAmmMar1_1/busco.

**Figure 2.**
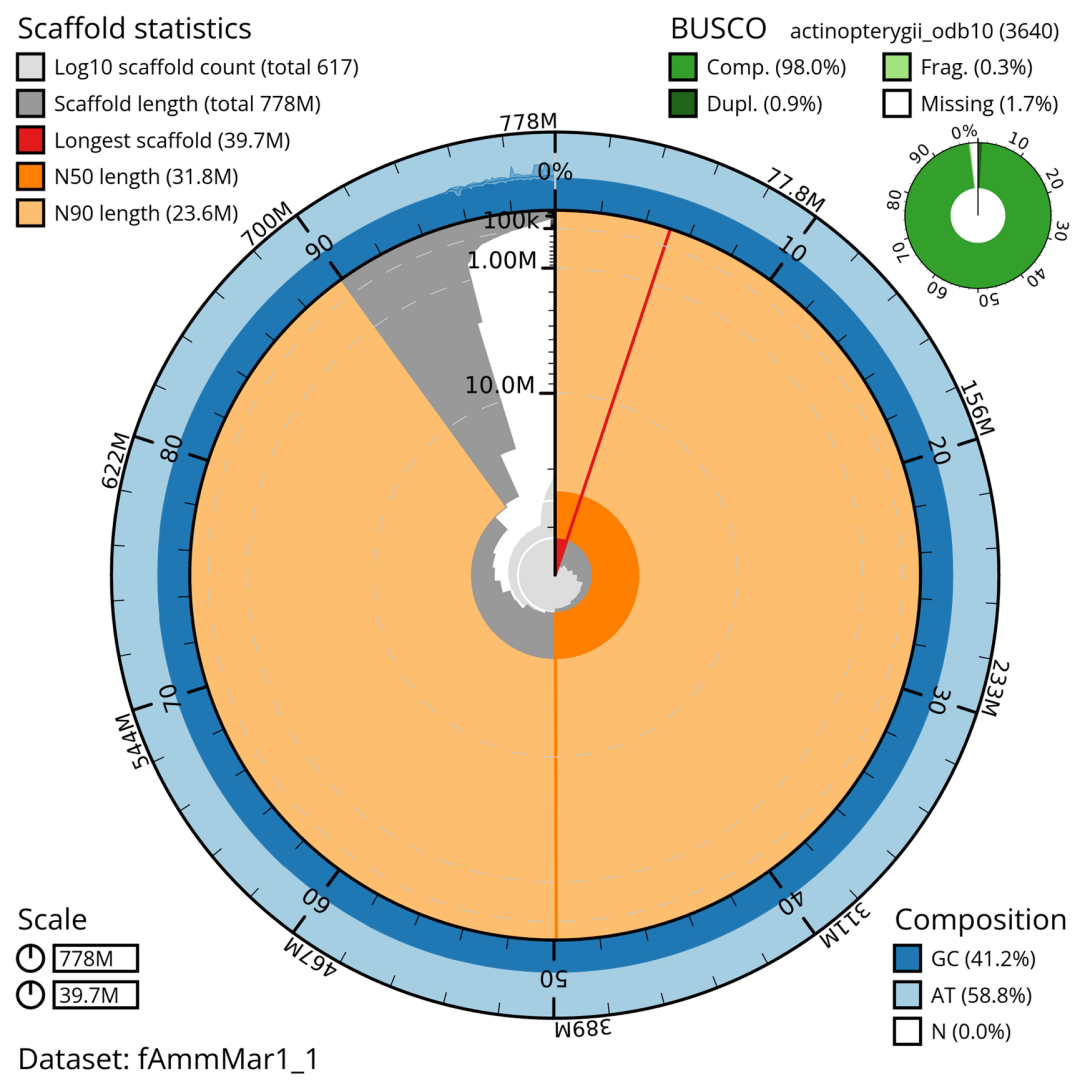
Genome assembly of *Ammodytes marinus*, fAmmMar1.1: metrics. The BlobToolKit snail plot shows N50 metrics and BUSCO gene completeness. The main plot is divided into 1,000 size-ordered bins around the circumference with each bin representing 0.1% of the 777,835,610 bp assembly. The distribution of scaffold lengths is shown in dark grey with the plot radius scaled to the longest scaffold present in the assembly (39,742,375 bp, shown in red). Orange and pale-orange arcs show the N50 and N90 scaffold lengths (31,846,471 and 23,554,204 bp), respectively. The pale grey spiral shows the cumulative scaffold count on a log scale with white scale lines showing successive orders of magnitude. The blue and pale-blue area around the outside of the plot shows the distribution of GC, AT and N percentages in the same bins as the inner plot. A summary of complete, fragmented, duplicated and missing BUSCO genes in the actinopterygii_odb10 set is shown in the top right. An interactive version of this figure is available at https://blobtoolkit.genomehubs.org/view/fAmmMar1_1/dataset/fAmmMar1_1/snail.

**Figure 3.**
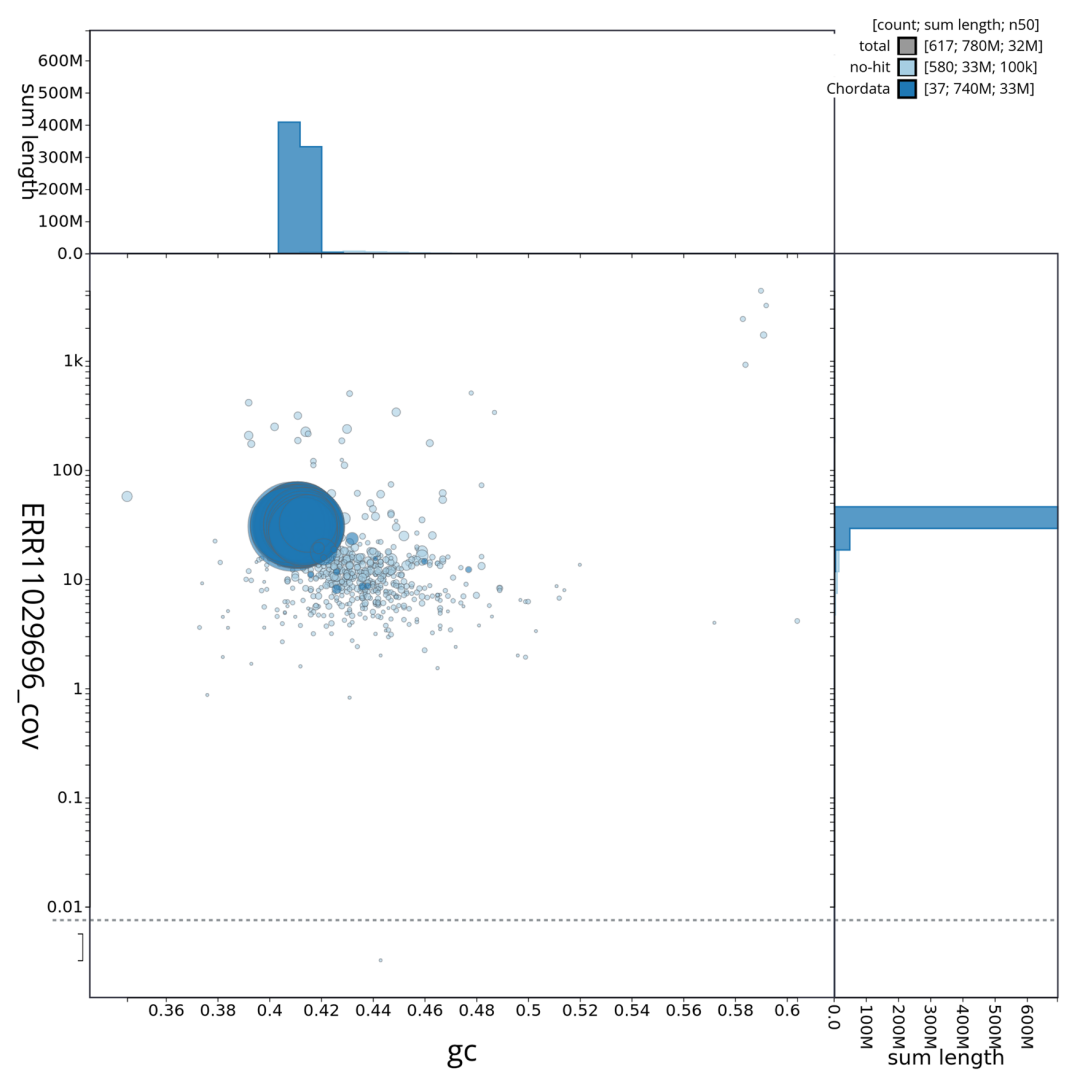
Genome assembly of *Ammodytes marinus*, fAmmMar1.1: BlobToolKit GC-coverage plot showing sequence coverage (vertical axis) and GC content (horizontal axis). The circles represent scaffolds, with the size proportional to scaffold length and the colour representing phylum membership. The histograms along the axes display the total length of sequences distributed across different levels of coverage and GC content. An interactive version of this figure is available at https://blobtoolkit.genomehubs.org/view/fAmmMar1_1/dataset/fAmmMar1_1/blob.

**Figure 4.**
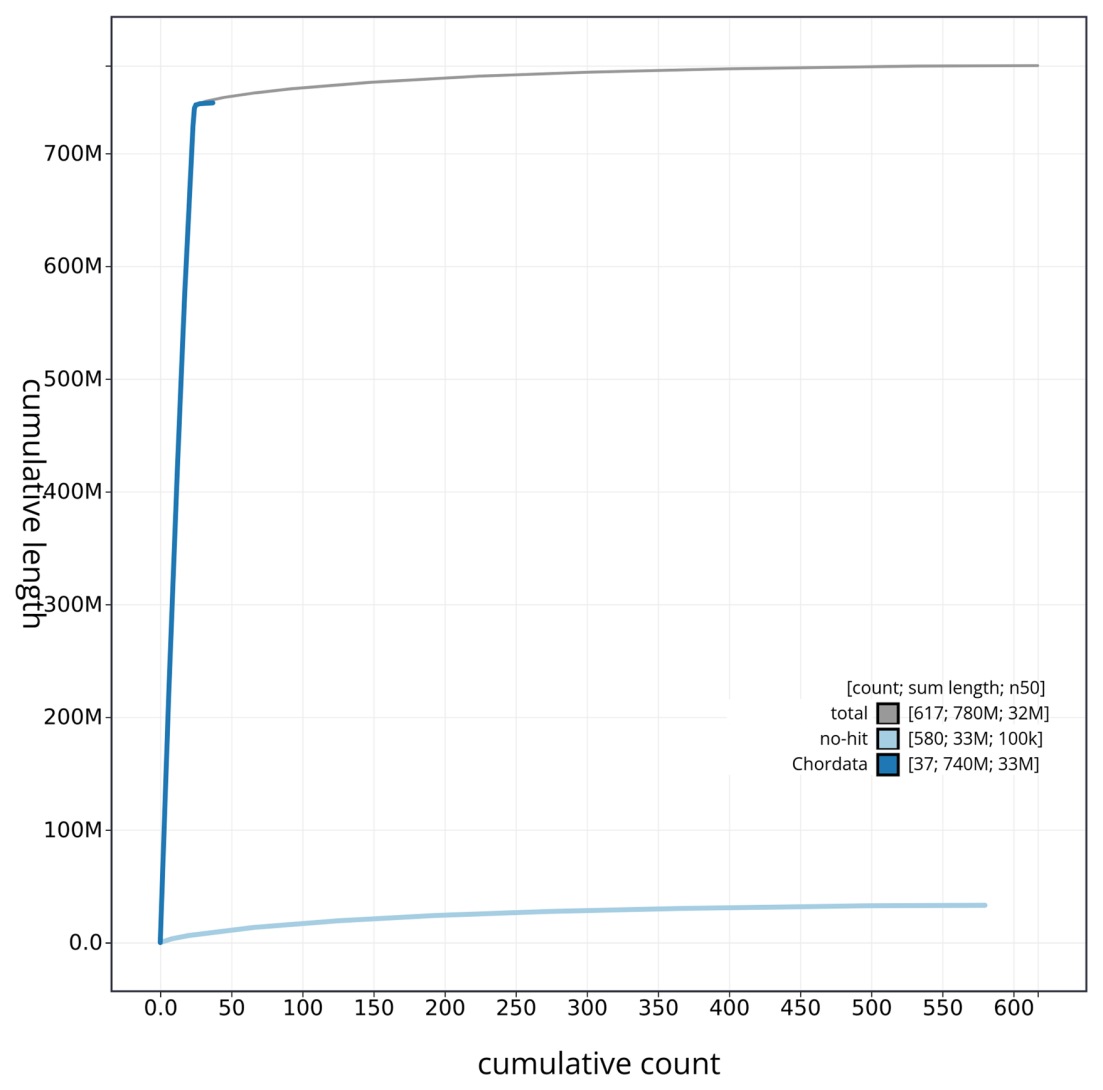
Genome assembly of *Ammodytes marinus* fAmmMar1.1: BlobToolKit cumulative sequence plot. The grey line shows cumulative length for all sequences. Coloured lines show cumulative lengths of sequences assigned to each phylum using the buscogenes taxrule. An interactive version of this figure is available at https://blobtoolkit.genomehubs.org/view/fAmmMar1_1/dataset/fAmmMar1_1/cumulative.

The snail plot in Figure 2 provides a summary of the assembly statistics, indicating the distribution of scaffold lengths and other assembly metrics. Figure 3 shows the distribution of scaffolds by GC proportion and coverage. Figure 4 presents a cumulative assembly plot, with separate curves representing different scaffold subsets assigned to various phyla, illustrating the completeness of the assembly.

**Figure 5.**
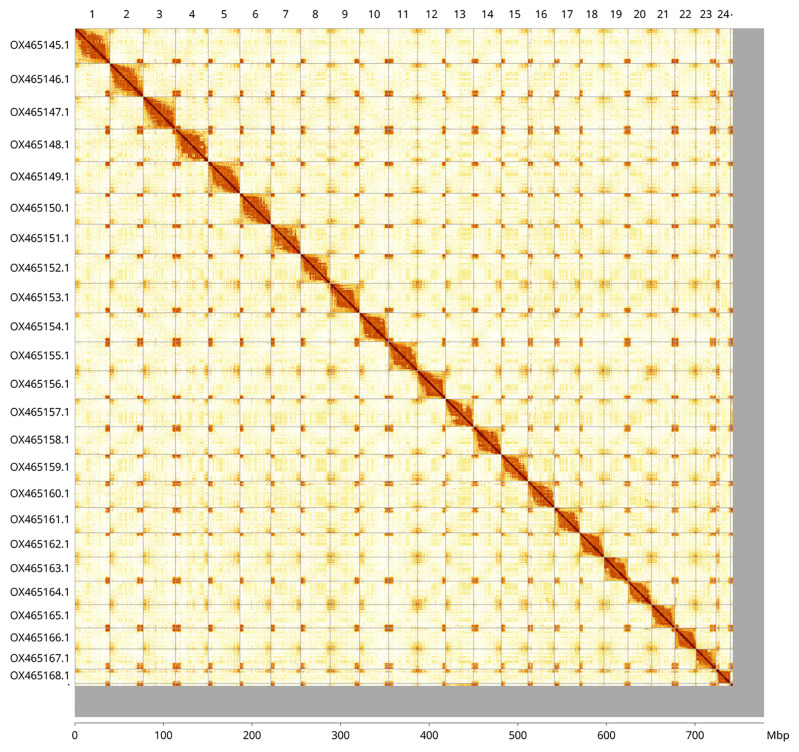
Genome assembly of *Ammodytes marinus* fAmmMar1.1: Hi-C contact map of the fAmmMar1.1 assembly, visualised using HiGlass. Assembled chromosomes are shown in order of size and labelled along the axes, with a megabase scale shown below. The plot was generated using PretextView and PretextSnapshot. An interactive version of this figure in HiGlass may be viewed at https://genome-note-higlass.tol.sanger.ac.uk/l/?d=G2gEVsC1TO2seAVccxAueQ.

Most of the assembly sequence (95.51%) was assigned to 25 chromosomal-level scaffolds. Chromosome-scale scaffolds confirmed by the Hi-C data are named in order of size ( Figure 5; Table 3). While not fully phased, the assembly deposited is of one haplotype. Contigs corresponding to an alternate haplotype have also been deposited.

**Table 3.** Chromosomal pseudomolecules in the genome assembly of *Ammodytes marinus*, fAmmMar1.

INSDC accession	Name	Length (Mb)	GC%
OX465145.1	1	39.74	41.0
OX465146.1	2	37.46	41.0
OX465147.1	3	36.71	41.0
OX465148.1	4	36.69	41.0
OX465149.1	5	35.78	41.0
OX465150.1	6	34.9	41.0
OX465151.1	7	33.56	41.0
OX465152.1	8	33.34	41.0
OX465153.1	9	33.22	41.0
OX465154.1	10	32.76	41.0
OX465155.1	11	32.61	41.0
OX465156.1	12	31.85	41.0
OX465157.1	13	31.42	41.0
OX465158.1	14	31.26	41.0
OX465159.1	15	30.11	41.5
OX465160.1	16	29.9	41.5
OX465161.1	17	28.48	41.5
OX465162.1	18	27.48	40.5
OX465163.1	19	27.02	41.0
OX465164.1	20	26.52	41.0
OX465165.1	21	26.51	41.5
OX465166.1	22	23.55	41.5
OX465167.1	23	22.99	41.5
OX465168.1	24	16.04	41.5
OX465169.1	25	3.03	42.0
OX465170.1	MT	0.02	48.0

The mitochondrial genome was also assembled and can be found as a contig within the multifasta file of the genome submission, and as a separate fasta file with accession OX465170.1 ( Table 2).

The final assembly has a Quality Value (QV) of 53.7. The *k*-mer completeness was estimated as 81.08% for the primary assembly, 79.48% for the alternate haplotype, and 98.50% for the combined assemblies. BUSCO (v5.4.3) analysis using the actinopterygii_odb10 reference set ( *n* = 3,640) achieved a completeness score of 98.1% (single = 97.1%, duplicated = 1.0%).

## Genome annotation report

The *Ammodytes marinus* genome assembly (GCA_949987685.1) was annotated at the European Bioinformatics Institute (EBI) on Ensembl Rapid Release. The resulting annotation includes 47,704 transcribed mRNAs from 22,410 protein-coding and 2,954 non-coding genes ( https://beta.ensembl.org/species/3711b4c9-2ac7-4e1d-9c30-3554f82bd875). The average transcript length is 18,875.56, with an average of 1.88 coding transcripts per gene and 12.03 exons per transcript.

## Methods

### Sample acquisition

An adult specimen of *Ammodytes marinus* (specimen ID ERGA SM FO 03, ToLID fAmmMar1) was collected aboard R/V Jákup Sverri at Faroe Bank (latitude 60.94, longitude –8.42) on 2021-09-10. The specimen was collected and identified by Helga Bára Mohr Vang (Faroe Marine Research Institute) using ( Mouritsen, 2007) which is based on ( Nelson *et al.*, 2016). Tissue samples were collected from the gills and fins and immediately snap frozen in liquid nitrogen by Ian Salter (Faroe Marine Research Institute) and stored at –80°C until nucleic acid extraction. The remaining portion of the specimen was preserved in 96% ethanol if further analyses were needed.

### Nucleic acid extraction

The workflow for high molecular weight (HMW) DNA extraction at the Wellcome Sanger Institute (WSI) Tree of Life Core Laboratory includes a sequence of core procedures: sample preparation and homogenisation, DNA extraction, fragmentation and purification. Detailed protocols are available on protocols.io ( Denton *et al.*, 2023b). The fAmmMar1 sample was prepared for DNA extraction by weighing and dissecting it on dry ice ( Jay *et al.*, 2023), and tissue from the gill was homogenised using a PowerMasher II tissue disruptor ( Denton *et al.*, 2023a).

HMW DNA was extracted using the Automated MagAttract v1 protocol ( Sheerin *et al.*, 2023). DNA was sheared into an average fragment size of 12–20 kb in a Megaruptor 3 system ( Todorovic *et al.*, 2023). Sheared DNA was purified by solid-phase reversible immobilisation, using AMPure PB beads to sample to eliminate shorter fragments and concentrate the DNA ( Strickland *et al.*, 2023). The concentration of the sheared and purified DNA was assessed using a Nanodrop spectrophotometer and Qubit Fluorometer using the Qubit dsDNA High Sensitivity Assay kit. Fragment size distribution was evaluated by running the sample on the FemtoPulse system.

RNA was extracted from fin tissue of fAmmMar1 in the Tree of Life Laboratory at the WSI using the RNA Extraction: Automated MagMax™ *mir*Vana protocol ( do Amaral *et al.*, 2023). The RNA concentration was assessed using a Nanodrop spectrophotometer and a Qubit Fluorometer using the Qubit RNA Broad-Range Assay kit. Analysis of the integrity of the RNA was done using the Agilent RNA 6000 Pico Kit and Eukaryotic Total RNA assay.

### Hi-C sample preparation

Tissue from the fin of specimen fAmmMar1 was processed at the WSI Scientific Operations core, using the Arima-HiC v2 kit. Tissue (stored at –80 °C) was fixed, and the DNA crosslinked using a TC buffer with 22% formaldehyde. After crosslinking, the tissue was homogenised using the Diagnocine Power Masher-II and BioMasher-II tubes and pestles. Following the kit manufacturer's instructions, crosslinked DNA was digested using a restriction enzyme master mix. The 5’-overhangs were then filled in and labelled with biotinylated nucleotides and proximally ligated. An overnight incubation was carried out for enzymes to digest remaining proteins and for crosslinks to reverse. A clean up was performed with SPRIselect beads prior to library preparation.

### Library preparation and sequencing

Library preparation and sequencing were performed at the WSI Scientific Operations core. Pacific Biosciences HiFi circular consensus DNA sequencing libraries were prepared using the PacBio Express Template Preparation Kit v2.0 (Pacific Biosciences, California, USA) as per the manufacturer's instructions. The kit includes the reagents required for removal of single-strand overhangs, DNA damage repair, end repair/A-tailing, adapter ligation, and nuclease treatment. Library preparation also included a library purification step using AMPure PB beads (Pacific Biosciences, California, USA) and size selection step to remove templates shorter than 3 kb using AMPure PB modified SPRI. DNA concentration was quantified using the Qubit Fluorometer v2.0 and Qubit HS Assay Kit and the final library fragment size analysis was carried out using the Agilent Femto Pulse Automated Pulsed Field CE Instrument and gDNA 165kb gDNA and 55kb BAC analysis kit. Samples were sequenced using the Sequel IIe system (Pacific Biosciences, California, USA). The concentration of the library loaded onto the Sequel IIe was between 40–135 pM. The SMRT link software, a PacBio web-based end-to-end workflow manager, was used to set-up and monitor the run, as well as perform primary and secondary analysis of the data upon completion.

For Hi-C library preparation, DNA was fragmented to a size of 400 to 600 bp using a Covaris E220 sonicator. The DNA was then enriched, barcoded, and amplified using the NEBNext Ultra II DNA Library Prep Kit following manufacturers’ instructions. The Hi-C sequencing was performed using paired-end sequencing with a read length of 150 bp on an Illumina NovaSeq 6000 instrument.

Poly(A) RNA-Seq libraries were constructed using the NEB Ultra II RNA Library Prep kit, following the manufacturer’s instructions. RNA sequencing was performed on the Illumina NovaSeq 6000 instrument.

### Genome assembly, curation and evaluation

***Assembly***

The HiFi reads were assembled using Hifiasm ( Cheng *et al.*, 2021) with the --primary option. Haplotypic duplications were identified and removed using purge_dups ( Guan *et al.*, 2020). The Hi-C reads were mapped to the primary contigs using bwa-mem2 ( Vasimuddin *et al.*, 2019). The contigs were further scaffolded using the provided Hi-C data ( Rao *et al.*, 2014) in YaHS ( Zhou *et al.*, 2023) using the --break option. The scaffolded assemblies were evaluated using Gfastats ( Formenti *et al.*, 2022), BUSCO ( Manni *et al.*, 2021) and MERQURY.FK ( Rhie *et al.*, 2020).

The mitochondrial genome was assembled using MitoHiFi ( Uliano-Silva *et al.*, 2023), which runs MitoFinder ( Allio *et al.*, 2020) and uses these annotations to select the final mitochondrial contig and to ensure the general quality of the sequence.

***Assembly curation***

The assembly was decontaminated using the Assembly Screen for Cobionts and Contaminants (ASCC) pipeline. Manual curation was primarily conducted using PretextView ( Harry, 2022), with additional insights provided by HiGlass ( Kerpedjiev *et al.*, 2018). Scaffolds were visually inspected and corrected as described by Howe *et al*. (2021). Any identified contamination, missed joins, and mis-joins were corrected, and duplicate sequences were tagged and removed. The entire process is documented at https://gitlab.com/wtsi-grit/rapid-curation. A Hi-C contact map of the final fasta file was produced using curationpretext and PretextSnapshot.

***Evaluation of the final assembly***

The Merqury.FK tool ( Rhie *et al.*, 2020), run in a Singularity container ( Kurtzer *et al.*, 2017), was used to evaluate *k*-mer completeness and assembly quality for the primary and alternate haplotypes using the *k*-mer databases ( *k* = 31) that were computed prior to genome assembly. The analysis outputs included assembly QV scores and completeness statistics.

A Hi-C contact map was produced for the final version of the assembly. The Hi-C reads were aligned using bwa-mem2 ( Vasimuddin *et al.*, 2019) and the alignment files were combined using SAMtools ( Danecek *et al.*, 2021). The Hi-C alignments were converted into a contact map using BEDTools ( Quinlan & Hall, 2010) and the Cooler tool suite ( Abdennur & Mirny, 2020). The contact map was visualised in HiGlass ( Kerpedjiev *et al.*, 2018).

The genome was analysed within the BlobToolKit environment ( Challis *et al.*, 2020) and BUSCO scores ( Manni *et al.*, 2021) were calculated.

Table 4 contains a list of relevant software tool versions and sources.

**Table 4.** Software tools: versions and sources.

Software tool	Version	Source
BEDTools	2.30.0	https://github.com/arq5x/bedtools2
BLAST	2.14.0	ftp://ftp.ncbi.nlm.nih.gov/blast/executables/blast+/
BlobToolKit	4.1.7	https://github.com/blobtoolkit/blobtoolkit
BUSCO	5.3.2	https://gitlab.com/ezlab/busco
bwa-mem2	2.2.1	https://github.com/bwa-mem2/bwa-mem2
Cooler	0.8.11	https://github.com/open2c/cooler
fasta_windows	0.2.4	https://github.com/tolkit/fasta_windows
FastK	1.1	https://github.com/thegenemyers/FASTK
GenomeScope2.0	2.0.1	https://github.com/tbenavi1/genomescope2.0
Gfastats	1.3.6	https://github.com/vgl-hub/gfastats
Hifiasm	0.16.1-r375	https://github.com/chhylp123/hifiasm
HiGlass	1.11.6	https://github.com/higlass/higlass
Merqury	1.1.2	https://github.com/thegenemyers/MERQURY.FK
MitoHiFi	3	https://github.com/marcelauliano/MitoHiFi
PretextView	0.2.5	https://github.com/wtsi-hpag/PretextView
purge_dups	1.2.5	https://github.com/dfguan/purge_dups
samtools	1.15.1	https://github.com/samtools/samtools
sanger-tol/ascc	0.1.0	https://github.com/sanger-tol/ascc
sanger-tol/curationpretext	1.4.2	https://github.com/sanger-tol/curationpretext
Seqtk	1.3	https://github.com/lh3/seqtk
Singularity	3.9.0	https://github.com/sylabs/singularity
YaHS	1.2a	https://github.com/c-zhou/yahs

### Genome annotation

The Ensembl Genebuild annotation system ( Aken *et al.*, 2016) was used to generate annotation for the *Ammodytes marinus* assembly (GCA_949987685.1) in Ensembl Rapid Release at the EBI. Annotation was created primarily through alignment of transcriptomic data to the genome, with gap filling via protein-to-genome alignments of a select set of proteins from UniProt ( UniProt Consortium, 2019).

### Wellcome Sanger Institute – Legal and Governance

The materials that have contributed to this genome note have been supplied by a Tree of Life collaborator.

The Wellcome Sanger Institute employs a process whereby due diligence is carried out proportionate to the nature of the materials themselves, and the circumstances under which they have been/are to be collected and provided for use. The purpose of this is to address and mitigate any potential legal and/or ethical implications of receipt and use of the materials as part of the research project, and to ensure that in doing so we align with best practice wherever possible.

The overarching areas of consideration are:

- Ethical review of provenance and sourcing of the material
- Legality of collection, transfer and use (national and international)

Each transfer of samples is undertaken according to a Research Collaboration Agreement or Material Transfer Agreement entered into by the Tree of Life collaborator, Genome Research Limited (operating as the Wellcome Sanger Institute) and in some circumstances other Tree of Life collaborators.

## Data Availability

European Nucleotide Archive: *Ammodytes marinus* (lesser sand-eel). Accession number PRJEB60704; https://identifiers.org/ena.embl/PRJEB60704. The genome sequence is released openly for reuse. The *Ammodytes marinus* genome sequencing initiative is part of the European Reference Genome Atlas Pilot Project ( https://www.erga-biodiversity.eu/pilot-project). All raw sequence data and the assembly have been deposited in INSDC databases. Raw data and assembly accession identifiers are reported in Table 1 and Table 2. Production code used in genome assembly at the WSI Tree of Life is available at https://github.com/sanger-tol.

## Author information

Members of the Wellcome Sanger Institute Tree of Life Management, Samples and Laboratory team are listed here: https://doi.org/10.5281/zenodo.12162482.

Members of Wellcome Sanger Institute Scientific Operations: Sequencing Operations are listed here: https://doi.org/10.5281/zenodo.12165051.

Members of the Wellcome Sanger Institute Tree of Life Core Informatics team are listed here: https://doi.org/10.5281/zenodo.12160324.

Members of the Tree of Life Core Informatics collective are listed here: https://doi.org/10.5281/zenodo.12205391.
