# Supplementary material for: The genome sequence of the lesser sand-eel, Ammodytes marinus Raitt, 1934
Source: Wellcome Open Res. 2025 Oct 6;10:113. Originally published 2025 Feb 28. [Version 2] doi: 10.12688/wellcomeopenres.23679.2 (PMC12770890; doi:10.12688/wellcomeopenres.23679.2)
Supplement: Peer review history

# Reviewer report: Reviewer response for version 2

**DOI:** 10.21956/wellcomeopenres.27595.r141679

**Published:** 2026-01-22

**Authors:** Madlen Stange, Temitope Opeyemi Oriowo

**Affiliations:** 1 Leibniz Institute for the Analysis of Biodiversity Change, Bonn, Germany; 2 Leibniz Institute for the Analysis of Biodiveristy Change, Germany, Germany

This manuscript has been co-reviewed by Temitope Opeyemi Oriowo.

 The data note report the genome assembly of the lesser eel, providing an assmebly and annotation for the nuclear genome as FASTA, GTF and GFF3 formats.  

 but not for the mitochondrial genome.

 Minor comments:

 1) a literature reference for sandeels being keystone species is missing. Since this is a very specific ecological concept, I would wish that the claim of sandeels being keystone species is not simply repeated but founded by evidence.

 Major comments:

 1) RNA sequencing: The usage of fin and just a single type of input material seems unusual. I was under the impression that it's best practice to use different organs as input for RNA sequencing for genome annotation. The annotation for this genome might be incomplete due to this.

 2) Nuclear genome annotation: Although there has been no specific mention of repeat annotation, I believe that 'genome annotation' also includes the annotation of repeats. No information has been provided on the type of repeats or the percentage of the genome consisting of repetitive elements, yet repeats have been annotated, as I can see both hard- and soft-masked assemblies. The (Aken et al., 2016) paper describes the various optional and essential methods used in this pipeline. For example, which RepeatMasker or RepeatModeler library is used? Which select set of proteins from UniProt, Vertebrates? While the data is accessible, the reproducibility of the methods is low.

 3) Mitochondrial genome annotation: I believe Mitohifi also outputs an annotation, not just an assembly. If you assembled the mitogenome, it is likely to be annotated but I can’t find any annotation for it, which however could be important for future phylogenetic studies including this species.

Are sufficient details of methods and materials provided to allow replication by others?

Partly

Is the rationale for creating the dataset(s) clearly described?

Yes

Are the datasets clearly presented in a useable and accessible format?

Yes

Are the protocols appropriate and is the work technically sound?

Yes

Reviewer Expertise:

T. Oriowo: genome assembly and annotation; M. Stange: fish genomics, whole genome sequencing, molecular biology

We confirm that we have read this submission and believe that we have an appropriate level of expertise to state that we do not consider it to be of an acceptable scientific standard, for reasons outlined above.

# Reviewer report: Reviewer response for version 2

**DOI:** 10.21956/wellcomeopenres.27595.r141674

**Published:** 2026-01-05

**Authors:** Sandra Lorena Ament-Velasquez

**Affiliations:** 1 Stockholm University, Stockholm, Sweden

This genome report presents a primary assembly and complementary haplotype contigs of an *Ammodytes marinus* individual, as well as its mitochondrial genome. An annotation of protein-coding genes is provided for the nuclear genome.

 I find the manuscript very complete and well-described. I confirmed that the primary and alternative assemblies, as well as the annotation, are available through the ENA and ENSEMBL websites.

 I have only two minor critiques:

 1) I saw no report on the presence of telomeric repeats, which could give a feeling of completion of the individual chromosomes, that is, how many and which chromosomes are fully assembled?

 2) The sex of the sequenced individual is not reported. In a reply to another reviewer, the authors stated that this is not relevant to the genome assembly report. However, I strongly disagree. Just knowing whether the individual is female or male may prove relevant for future research on sex determination. If the sex is unknown, this should be stated anyway.

 I do not have knowledge on the taxonomy, ecology, or economic importance of this fish so I cannot comment on the accuracy of its description.

Are sufficient details of methods and materials provided to allow replication by others?

Yes

Is the rationale for creating the dataset(s) clearly described?

Yes

Are the datasets clearly presented in a useable and accessible format?

Yes

Are the protocols appropriate and is the work technically sound?

Yes

Reviewer Expertise:

Evolutionary genomics

I confirm that I have read this submission and believe that I have an appropriate level of expertise to confirm that it is of an acceptable scientific standard.

# Reviewer report: Reviewer response for version 2

**DOI:** 10.21956/wellcomeopenres.27595.r135121

**Published:** 2025-11-06

**Authors:** Khairul Syahputra

**Affiliations:** 1 Research Center for Freshwater Aquaculture, National Research and Innovation Agency (BRIN), Cibinong, Indonesia

The revised manuscript has been significantly improved based on the previous comments. The current form of manuscript is suitable for indexing in my perspective!

Are sufficient details of methods and materials provided to allow replication by others?

Partly

Is the rationale for creating the dataset(s) clearly described?

Yes

Are the datasets clearly presented in a useable and accessible format?

Yes

Are the protocols appropriate and is the work technically sound?

Yes

Reviewer Expertise:

Fish genetic, WGS, RNAseq, Aquaculture

I confirm that I have read this submission and believe that I have an appropriate level of expertise to confirm that it is of an acceptable scientific standard.

# Reviewer report: Reviewer response for version 2

**DOI:** 10.21956/wellcomeopenres.27595.r135122

**Published:** 2025-11-03

**Authors:** Artur Burzynski

**Affiliations:** 1 Institute of Oceanology, Sopot, Poland

The changes are only relevant for the minor points raised.

 The main issue of not sharing or even performing adequate annotation/verification of the end results remains.

 If the pipeline is inadequate, then it should be fixed. The suggestion that the interested reader can do all the annotations/verification at will, given that the raw data are shared, is not acceptable. If the sole purpose of this data note is to share raw data and metadata, then I do not see any reason to review it: it is a trivial, technical listing. I do not think anybody looking e.g. for barcoding COX1 of this species or a specific rDNA marker as part of a larger study, will have the resources, time and awareness, that even though this is essentially done and available, it is hidden from the public because the authors did not publicize their results in any of the official INSDB databases.

Are sufficient details of methods and materials provided to allow replication by others?

Partly

Is the rationale for creating the dataset(s) clearly described?

Yes

Are the datasets clearly presented in a useable and accessible format?

Partly

Are the protocols appropriate and is the work technically sound?

Yes

Reviewer Expertise:

Mitogenomics, genetics and transcriptomics of marine organisms.

I confirm that I have read this submission and believe that I have an appropriate level of expertise to state that I do not consider it to be of an acceptable scientific standard, for reasons outlined above.

# Reviewer report: Reviewer response for version 1

**DOI:** 10.21956/wellcomeopenres.26122.r120525

**Published:** 2025-04-18

**Authors:** Khairul Syahputra

**Affiliations:** 1 Research Center for Freshwater Aquaculture, National Research and Innovation Agency (BRIN), Cibinong, Indonesia

Comments WOR – genome the lesser sand-eel

 Manuscript is well prepared, and the genome information of present study is interesting and would be useful for genetic studies of aquatic animals, particularly fishes. However, the following minor issues need to be addressed before manuscript is acceptable.

 The common name of species should be used to mention the species throughout the text, the Latin name is only included in the first appearance of the species in the text.

 Background:

 Figure 1 should be improved. I would recommend using the original image from the present study (if possible, image of the specimen).

 Methods:

 In sample acquisition, the authors did not explain how tissue sample for RNA extraction was collected and stored. State this!

Are sufficient details of methods and materials provided to allow replication by others?

Partly

Is the rationale for creating the dataset(s) clearly described?

Yes

Are the datasets clearly presented in a useable and accessible format?

Yes

Are the protocols appropriate and is the work technically sound?

Yes

Reviewer Expertise:

Fish genetic, WGS, RNAseq

I confirm that I have read this submission and believe that I have an appropriate level of expertise to confirm that it is of an acceptable scientific standard, however I have significant reservations, as outlined above.

# Response

**Published:** 2025-09-26

**Authors:** Tree of Life Team Sanger

**Affiliations:** Wellcome Trust Sanger Institute, UK

Thank you for reviewing this data note.   

 1.    The common name of species should be used to mention the species throughout the text; the Latin name is only included in the first appearance of the species in the text.

***Response: ** Thank you for the suggestion. For data notes we use the scientific (Latin) name throughout, after giving the common name at first mention. The article focuses on a specific sequenced specimen and its genome; consistent use of the binomial reduces ambiguity.*  

 2.    Figure 1 should be improved. I would recommend using the original image from the present study (if possible, image of the specimen).

***Response:** No photo was taken of the original specimen. We have replaced the image with a photo of an Ammodytes marinus individual sampled by the Faroe Marine Research Institute, but was not used in this study.*  

 3.    Methods: In sample acquisition, the authors did not explain how tissue sample for RNA extraction was collected and stored. State this. 

***Response:** The sampling method has now been expanded to include the collection of the fin sample. *  

 The Tree of Life team

# Reviewer report: Reviewer response for version 1

**DOI:** 10.21956/wellcomeopenres.26122.r119937

**Published:** 2025-03-24

**Authors:** Artur Burzynski

**Affiliations:** 1 Institute of Oceanology, Sopot, Poland

The article is adequately summarized in the Abstract.

 Missing elements are:

 1). The species reference image is not original, therefore it is unclear if the sequenced material comes from this species. It would be better to have the image of the sampled specimen instead. More details on the species identification procedure should be given (why do you think the sampled material comes from the target species?). A specific reference to a taxonomic key used in this procedure should also be given, so that this is replicable, not just a name of the researcher who did this work  Why the morphological parameters which were "recorded" are not reported here?

 2). The annotations. Mitogenome is not annotated at all. The other annotations which can be viewed in the records listed in Table 2 constitute only mapping between the assembled genome and the RNA-seq data. Genes are NOT specifically identified. This is strange, given that both the referenced tools listed under "GEnome annotation" should provide specific references for genes. Perhaps this is a matter of data propagation or unclear referencing in the manuscript or some kind of mess up in the databases, but without these results readily available, the reported assembly is far less relevant than it should be.

 3). Not all tools are acknowledged in all pipelines. I do realize this is quickly getting very complicated, but at least some of the tools internally used by the pipeline-only software pieces (such as e.g. MitoHiFi or BUSCO), do have e.g. CC By 4.0  licenses -  which require their attribution. And even if the licenses are different, the fir use policy dictates to acknowledge the work on which we build. In short: all underlying tools should be acknowledged, this should be checked throughout the contents of Table 4, which should be augmented with both rows (listing these tools) and a column (with paper references, whenever possible).

Are sufficient details of methods and materials provided to allow replication by others?

Partly

Is the rationale for creating the dataset(s) clearly described?

Yes

Are the datasets clearly presented in a useable and accessible format?

Partly

Are the protocols appropriate and is the work technically sound?

Yes

Reviewer Expertise:

Mitogenomics, genetics and transcriptomics of marine organisms.

I confirm that I have read this submission and believe that I have an appropriate level of expertise to confirm that it is of an acceptable scientific standard, however I have significant reservations, as outlined above.

# Response

**Published:** 2025-09-26

**Authors:** Tree of Life Team Sanger

**Affiliations:** Wellcome Trust Sanger Institute, UK

Thank you for reviewing this data note.

 1) The species reference image is not original; therefore, it is unclear if the sequenced material comes from this species. It would be better to have the image of the sampled specimen instead. More details on the species identification procedure should be given (why do you think the sampled material comes from the target species?). A specific reference to a taxonomic key used in this procedure should also be given, so that this is replicable, not just a name of the researcher who did this work Why the morphological parameters which were "recorded" are not reported here?

**Response:** *No photo was taken of the original specimen. We have replaced the image with a photo of an *Ammodytes marinus *individual sampled by the Faroe Marine Research Institute, but was not used in this study.* *Length, weight, life stage, and sex was not reported because it isn’t relevant for the genome assembly. The sentence “*The following biological parameters were recorded for the specimen: length, weight, life stage, and sex. *” Has now been removed. The method has been updated to include the identification key used.*  

 2) The annotations. Mitogenome is not annotated at all. The other annotations which can be viewed in the records listed in Table 2 constitute only mapping between the assembled genome and the RNA-seq data. Genes are NOT specifically identified. This is strange, given that both the referenced tools listed under "GEnome annotation" should provide specific references for genes. Perhaps this is a matter of data propagation or unclear referencing in the manuscript or some kind of mess up in the databases, but without these results readily available, the reported assembly is far less relevant than it should be.

***Response: **The genome annotation is provided externally by Ensembl at the European Bioinformatics Institute. We have provided a link (* https://beta.ensembl.org/species/3711b4c9-2ac7-4e1d-9c30-3554f82bd875) *to the site where the fasta, gtf and gff3 files can be downloaded. Unfortunately, we do not provide annotation of the mitogenome as part of our pipeline, but the sequence may be downloaded and annotated.*

 3) Not all tools are acknowledged in all pipelines. I do realize this is quickly getting very complicated, but at least some of the tools internally used by the pipeline-only software pieces (such as e.g. MitoHiFi or BUSCO), do have e.g. CC By 4.0 licenses - which require their attribution. And even if the licenses are different, the fir use policy dictates to acknowledge the work on which we build. In short: all underlying tools should be acknowledged, this should be checked throughout the contents of Table 4, which should be augmented with both rows (listing these tools) and a column (with paper references, whenever possible).

***Response:** We have expanded table 4 with additional rows. Paper references are given as in-text citations. We have also provided a link in the Data availability section. *  

 The Tree of Life team
